# The utilization of efgartigimod in the treatment of acute cerebellar ataxia: a case report

**DOI:** 10.3389/fimmu.2025.1581954

**Published:** 2025-08-27

**Authors:** Liu L. Yang, Yushuang Ruan, Fucheng Cai, Yuanpeng Xia

**Affiliations:** ^1^ Department of Neurology, Union Hospital, Tongji Medical College, Huazhong University of Science and Technology, Wuhan, China; ^2^ Department of Pediatrics, Union Hospital, Tongji Medical College, Huazhong University of Science and Technology, Wuhan, China

**Keywords:** acute cerebellar ataxia, autoimmune, antibody, efgartigimod, neonatal Fc receptor, children

## Abstract

**Background:**

Acute cerebellar ataxia (ACA) is the most common cause of acute ataxia in children and adolescents. It is a cerebellar disorder with multifactorial pathogenesis, often triggered by viral or bacterial infections, as well as autoimmune mechanisms. The clinical course of ACA can vary widely, ranging from a benign, self-limiting condition to a severe, life-threatening illness. There is no universally accepted consensus on the optimal management of ACA in children. While steroids and immunoglobulins are commonly used, some patients may not respond adequately to these treatments. Efgartigimod, a novel immune modulator, has not been previously reported in the treatment of ACA, but its unique mechanism of action suggests potential therapeutic benefits.

**Case presentation:**

We present a case of an 11-year-old girl with ACA who was treated with efgartigimod and showed significant improvements. The patient presented with acute ataxia, slurred speech, vomiting, diarrhea, abdominal pain, dizziness, and altered mental status. Initial investigations, including routine blood tests, specific autoantibodies related to cerebellitis in both serum and cerebrospinal fluid (CSF), and brain magnetic resonance imaging (MRI) revealed no abnormalities. Despite treatment with neurotrophic drugs and dexamethasone, the patient showed minimal improvement. A trial of efgartigimod (10 mg/kg) resulted in rapid symptom alleviation within three days.

**Conclusion:**

This case highlights the potential role of efgartigimod in the treatment of ACA, particularly in cases refractory to conventional therapies. Further studies are needed to validate the efficacy and safety of Efgartigimod in pediatric patients with ACA.

## Introduction

Acute cerebellar ataxia (ACA) is an immune-mediated syndrome characterized by the acute onset of cerebellar-associated neurological signs, such as ataxia, nystagmus, and dysmetria, often accompanied by systemic symptoms like fever, nausea, headache, and altered mental status (1). The condition is most prevalent in children and adolescents and is frequently associated with post-infectious autoimmune mechanisms following viral or bacterial infections (2). Diagnosis is typically supported by neuroimaging, cerebrospinal fluid (CSF) analysis, and the presence of specific autoantibodies, though some cases may present with negative findings, complicating the diagnostic process (2).

The management of ACA remains challenging, with no established consensus on the optimal treatment approach (3). While most cases resolve with supportive care, severe or refractory cases may require immunomodulatory therapies such as steroids or intravenous immunoglobulins (IVIG) (4). Efgartigimod, a neonatal Fc receptor (FcRn) inhibitor, has shown promise in the treatment of immunoglobulin G (IgG)-mediated autoimmune diseases, including myasthenia gravis (MG) and chronic inflammatory demyelinating polyradiculoneuropathy (CIDP) (5, 6). Its mechanism of action involves reducing circulating IgG levels, including pathogenic autoantibodies, thereby modulating the immune response (5). Based on the mechanism of rapidly clearing antibodies from the serum, it may demonstrate significant therapeutic efficacy in the acute phase of immune-mediated cerebellar ataxia.

## Case presentation

An 11-year-old girl presented to the pediatric emergency department with a 10-day history of severe ataxia, and unclear speech. Over the preceding two days, she developed additional symptoms, including vomiting, watery diarrhea, abdominal pain, dizziness, and mental sluggishness (manifested as slowed responses and reduced speech output). The patient had experienced a mild cold two weeks prior, which resolved spontaneously. She had her menarche five days prior to admission. There was no significant medical or family history of neurological disorders.

Upon admission, the neurological examination revealed normal vital signs (temperature: 36.5 °C, heart rate: 96 bpm, respiratory rate: 20/min, blood pressure: 119/76 mmHg). The patient was conscious with an unremarkable general appearance. Neurological deficits were significant, including slowed responses and dysarthria characterized by reduced volume, unclear articulation, and explosive speech. Cranial nerve function was intact. While muscle bulk and tone were normal in all four limbs, with preserved voluntary movement, strength was reduced in the lower limbs (Medical Research Council grade 4). Sensory examination was normal except for impaired vibratory sensation in the left toes. Reflexes were normoactive in the upper limbs but hyperreflexic in the lower limbs, with no pathological signs present. Cerebellar testing demonstrated bilateral dysmetria on finger-nose, heel-shin, and rapid alternating movement tests. Posture was normal, but gait was slow with a positive Romberg sign. Meningeal signs (neck stiffness, Kernig’s sign, Brudzinski’s sign) and the straight-leg-raise test (Lasègue’s sign) were negative. A Scale for the Assessment and Rating of Ataxia (SARA) score of 18 confirmed significant cerebellar dysfunction (see **Supplementary Table 1**). Consistent with hospital protocol at the time, this examination was not recorded on video.

Common biological investigations revealed normal hemoglobin concentration (121 g/L), platelet count of 362*10^9^/L, C-reactive protein level (0.27 mg/L) and slightly abnormal white blood cell count (with 71.1% neutrophils ↑ and 22.5% lymphocytes ↓). Serological liver and kidney function, blood lipid levels were normal, with a slightly increased fasting glucose level (6.2 mmol/L) and decreased potassium level (3.44 mmol/L) (**Table 1**). Infectious screening was conducted, encompassing serological analyses for cytomegalovirus and toxoplasmosis, both of which yielded negative results for IgM and IgG antibodies. Additionally, virological testing was performed to confirm Epstein-Barr virus (EBV) infection. The results indicated negativity for the EBV nuclear antigen but positivity for both IgG and IgM viral capsid antigens.

**Table 1:** Initial serologic.

Test	Result	Reference range (unit)
Routine		
White blood cell	7.29	4.3-11.3 (*10^9^/L)
Red blood cell	4.29	4.3-5.8 (*10^12^/L)
Neutrophile granulocyte	5.19	1.6-7.8 (*10^9^/L)
Eosinophilic granulocyte	2.4	0.0-9 (*10^9^/L)
Basophilic granulocyte	0.1	0.0-1 (*10^9^/L)
Lymphocyte	1.64	1.5-4.6 (*10^9^/L)
Platelet	362	167-453 (*10^9^/L)
Hemoglobin	121	118-156 (g/L)
C-reactive protein	0.27	0-5 (mg/L)
Total protein	68.7	65-84 (g/L)
Albumin	44.1	39-54 (g/L)
Globulin	24.6	18-38 (g/L)
Aspartate aminotransferase	<10	7-30 (U/L)
Alanine aminotransferase	18	14-44 (U/L)
Uric acid	259.8	155-357 (μmol/L)
Creatinine	37.2	27-66 (μmol/L)
Urea nitrogen	3	2.5-6.5 (mmol/L)
Glomerular filtration rate	157.59	90-120 (ml/min)
Sodium	140.5	135-145 (mmol/L)
Potassium	3.44 ↓	3.7-5.2 (mmol/L)
Chlorine	104.9	98-110 (mmol/L)
Fasting glucose	6.2 ↑	3.9-6.1 (mmol/L)
Triglyceride	0.89	0-1.7 (mmol/L)
Total cholesterol	4.69	0-5.2 (mmol/L)
Low density lipoprotein cholesterol	2.82	2.7-3.1 (mmol/L)
High density lipoprotein cholesterol	1.32	1.29-1.55 (mmol/L)
Creatine kinase	50	26-140 (U/L)
Tumor maker		
Human epididymal protein 4	31.5	<70 (pmol/L)
Alpha-fetoprotein	1.8	0.89-8.78 (μg/L)
Carcinoembryonic antigen	1	<5 (μg/L)
Carbohydrate antigen 125	20.5	<35 (U/ml)
Carbohydrate antigen 19-9	23.3	<37 (U/ml)
Carbohydrate antigen 15-3	10.8	<31.3 (U/ml)
Carbohydrate antigen 72-4	1.19	<6.9 (U/ml)
Human chorionic gonadotropin-beta	<1.2	<5 (IU/L)
Squamous cell carcinoma-antigen	0.5	<1.5 (ng/ml)
Cytokeratin 19 Fragment	0.79	<2.5 (ng/ml)
Neuron-specific enolase	15.93	<16.3 (μg/ml)
Ferritin	48.1	4.6-204 (μg/ml)
Autoimmune		
Anti-RNPA antibody titer	<1:100	
Anti-RNP68 antibody	<0.2	
Anti-Sm/RNP antibody	<0.2	
Anti-Sm antibody	<0.2	
Anti-SS-A antibody	<0.2	
Anti-Ro-52 antibody	<0.2	
Anti-SS-B antibody	<0.2	
Anti-SCI70 antibody	<0.2	
Anti JO-1 antibody	<0.2	
Anti-dsDNA antibody	1	<10 (IU/ml)
Anti-ANA antibody	<0.2	
Anti-ribosomal p protein	<0.2	
Anti-chromatin antibody	<0.2	
Immunoglobulin G	10.5	7.51-15.6 (g/L)
Immunoglobulin M	0.739	0.46-3.04 (g/L)
Immunoglobulin A	0.88	0.82-4.53 (g/L)
Immunoglobulin E	95	1-190 (IU/ml)
Complement 3	0.778	0.79-1.52 (g/L)
Complement 5	0.151	0.16-0.38 (g/L)

"↓" means decrease, and "↑" means increase.

**Figure 1:**
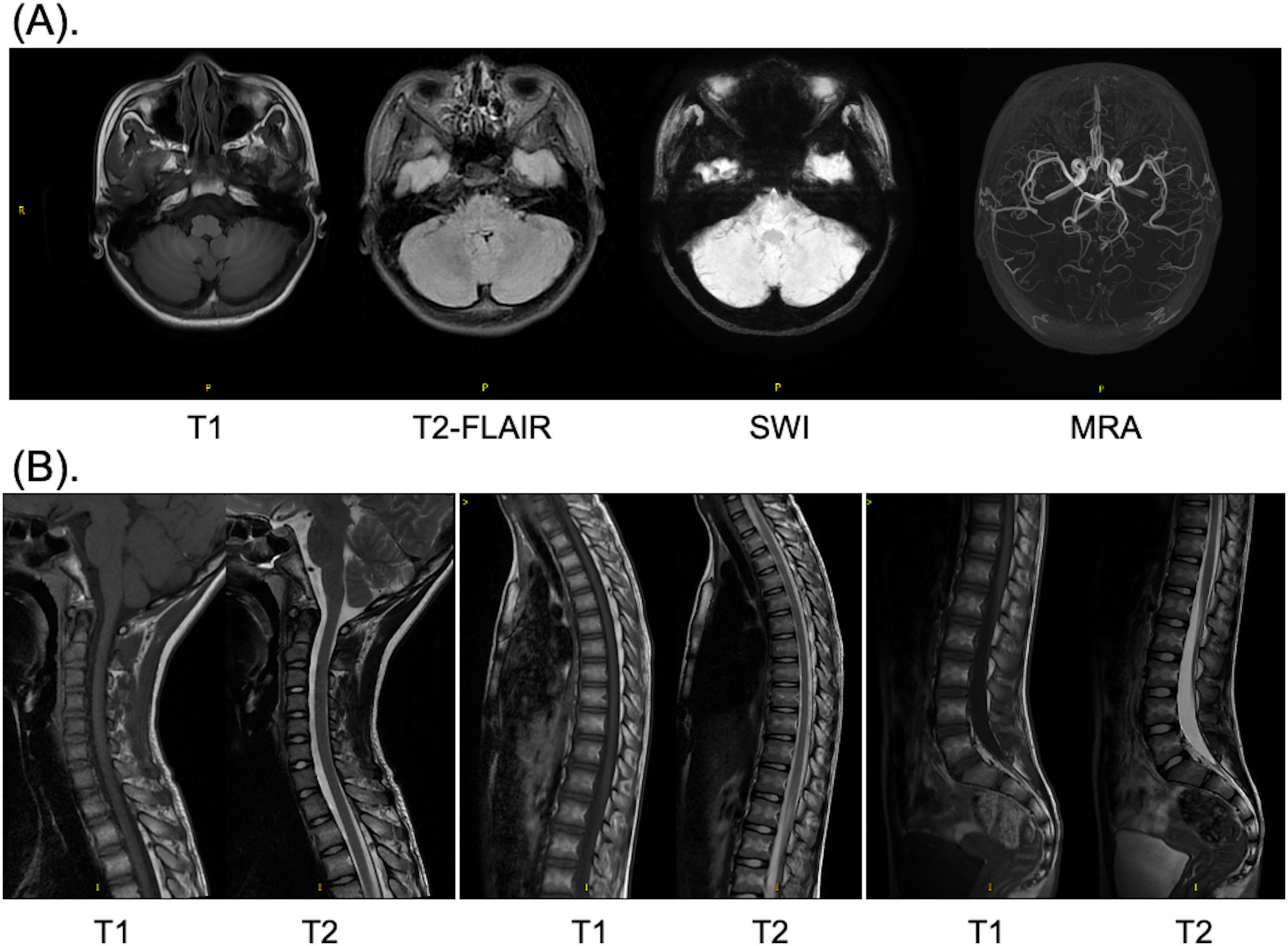
MRI images of the brain **(A)** and whole spine **(B)** after admission. **(A)** T1 weighted, T2 weighted, SWI and MRA scan of the brain; **(B)** T1 and T2 weighted scan of the cervical, thoracic, and lumbar vertebrae. MRI, magnetic resonance imaging; SWI, susceptibility weighted imaging; MRA, magnetic resonance angiography.

**Figure 2:**
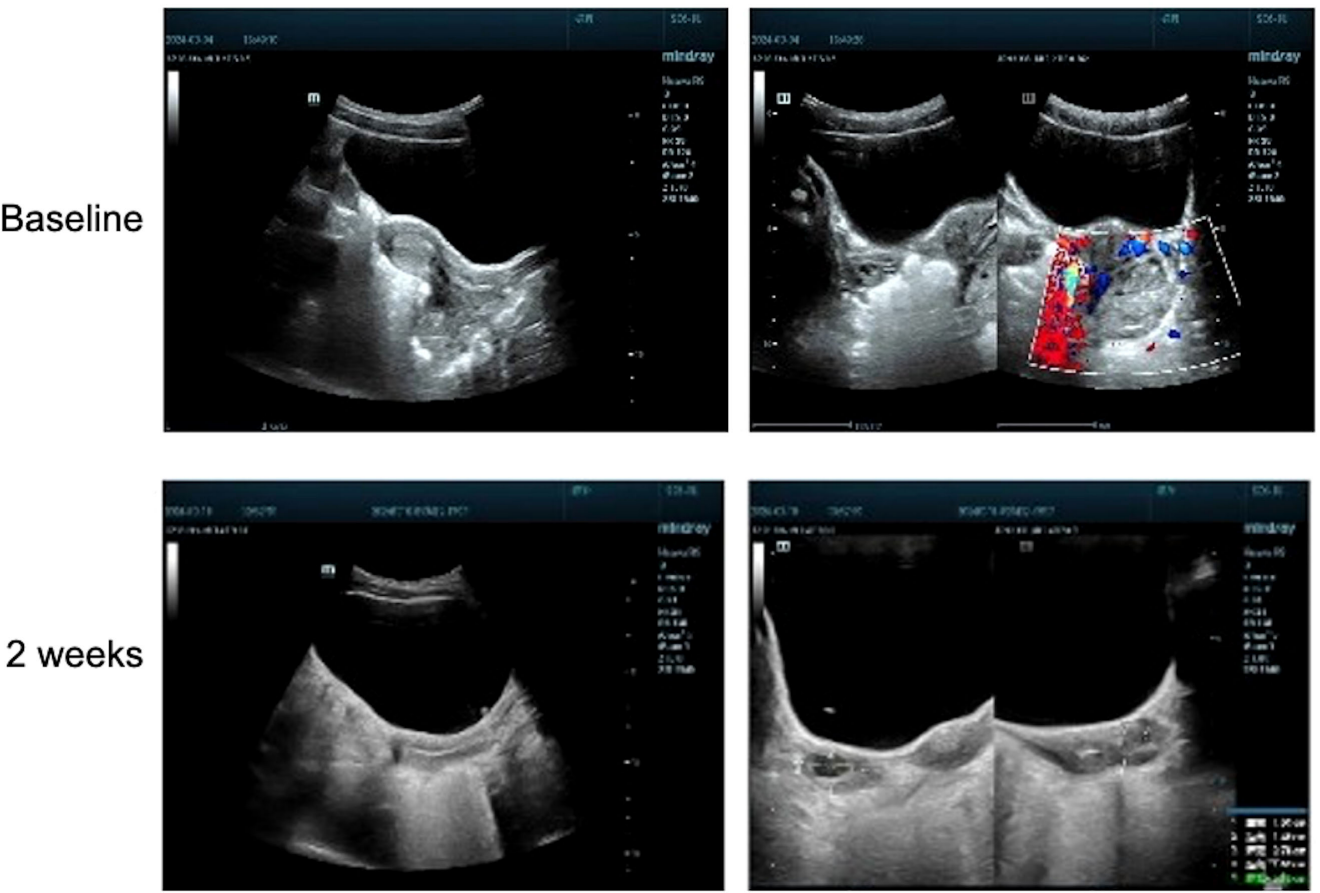
Gynecological ultrasound images acquired before (baseline) and after (2 weeks) administration of efgartigimod.

The brain computed tomography scan and electroencephalogram (EEG) revealed no abnormalities (**Supplementary Figure 1**). Subsequently, a comprehensive magnetic resonance imaging (MRI) was conducted to differentiate between spinal pathologies and acute cerebellitis. Both the brain MRI and whole spine MRI demonstrated no pathological findings (**Figure 1**). Notably, no evidence of hemorrhage was observed on susceptibility-weighted imaging sequences, and there was no indication of diffusion restriction (**Figure 1**). Unexpectedly, upon imaging the left adnexal region, a cystic lesion measuring approximately 20 mm was identified. Further evaluation with gynecological ultrasound revealed a left ovarian lesion with uniform echogenicity, measuring 4.0 × 3.5 × 3.1 cm, the nature of which remains to be elucidated (**Figure 2**). To rule out paraneoplastic syndrome, serum tumor markers specific to females were analyzed, all of which were within normal limits (**Table 1**).

In order differentiate from Guillain-Barré syndrome (GBS) and the spectrum disorders [including Miller-Fisher syndrome (MFS), Bickerstaff’s brainstem encephalitis (BBE)] and acute disseminated encephalomyelitis, a diagnostic lumbar puncture was successfully performed on the third day after admission. CSF analysis demonstrated no evidence of intracranial hypertension, with an opening pressure of 120 mmH_2_O. The CSF protein level was measured at 0.11 g/L, and the glucose level was 4.01 mmol/L, with a corresponding serum glucose level of 6.2 mmol/L (**Supplementary Table 2**). No albuminocytolgoic dissociation and pleocytosis were observed. To further investigate potential peripheral neuropathy, 12 anti-ganglioside antibodies and 7 Ranvier node-associated antibodies were also tested, none of which were definite positive (**Table 2**). An electromyography was performed, but failed to obtain effective results, due to poor cooperation of the patient. Comprehensive infectious screening, including tests for common bacteria, fungi, Mycobacterium tuberculosis, and Cryptococcus neoformans, yielded negative results (**Supplementary Table 2**). Acute intracranial infections were successfully excluded. Furthermore, an extensive panel of 16 specific antibodies associated with autoimmune cerebellitis was analyzed in both serum and CSF, all of which were negative (**Table 2**). Additionally, a complete panel of autoimmune disease associated antibodies in serum was also tested, with no abnormalities detected (**Table 1**). Despite the absence of positive biological findings, the patient was strongly suspected to have autoimmune ACA based on the clinical presentations and history of pre infection alone.

**Table 2:** Antibody tests of autoimmune cerebellitis panel, anti-ganglioside panel and Ranvier node-associated panel.

Test	Detection	Result	CSF
	method	Serum	
Autoimmune cerebellitis panel			
anti-Hu IgG	ELISA	negative (-)	negative (-)
anti-Yo IgG	ELISA	negative (-)	negative (-)
anti-CV2 IgG	ELISA	negative (-)	negative (-)
anti-Ma2 IgG	ELISA	negative (-)	negative (-)
anti-Amphiphysin IgG	ELISA	negative (-)	negative (-)
anti-Tr(DNER) IgG	ELISA	negative (-)	negative (-)
anti-Zic4 IgG	ELISA	negative (-)	negative (-)
anti-Ma1 IgG	ELISA	negative (-)	negative (-)
anti-SOX1 IgG	ELISA	negative (-)	negative (-)
anti-PKC𝛾 IgG	ELISA	negative (-)	negative (-)
anti-Ri IgG	ELISA	negative (-)	negative (-)
anti-Homer3 IgG	CBA	negative (-)	negative (-)
anti-ATP1A3 IgG	CBA	negative (-)	negative (-)
anti-ARHGAP2 IgG	CBA	negative (-)	negative (-)
anti-ITPR1 IgG	CBA	negative (-)	negative (-)
anti-GAD65 IgG	CBA	negative (-)	negative (-)
Anti-ganglioside panel			
anti-sulfatides IgG	CBA	negative (-)	negative (-)
anti-GM1 IgG	CBA	negative (-)	negative (-)
anti-GM2 IgG	CBA	negative (-)	negative (-)
anti-GM3 IgG	CBA	negative (-)	negative (-)
anti-GM4 IgG	CBA	suspicious (+/-)	negative (-)
anti-GD1a IgG	CBA	suspicious (+/-)	negative (-)
anti-GD1b IgG	CBA	negative (-)	negative (-)
anti-GD2 IgG	CBA	negative (-)	negative (-)
anti-GD3 IgG	CBA	negative (-)	negative (-)
anti-GT1a IgG	CBA	suspicious (+/-)	negative (-)
anti-GT1b IgG	CBA	negative (-)	negative (-)
anti-GQ1b IgG	CBA	negative (-)	negative (-)
Ranvier node-associated panel			
anti-NF155 IgG	CBA	negative (-)	negative (-)
anti-NF186 IgG	CBA	negative (-)	negative (-)
anti-CASPR1 IgG	CBA	negative (-)	negative (-)
anti-CASPR2 IgG	CBA	negative (-)	negative (-)
anti-CNTN1 IgG	CBA	negative (-)	negative (-)
anti-CNTN2 IgG	CBA	negative (-)	negative (-)
anti-MAG IgG	CBA	negative (-)	negative (-)

The 16 autoimmune cerebellitis antibodies, 12 anti-ganglioside antibodies and 7 Ranvier node associated antibodies in both serum and cerebrospinal fluid (CSF). The results were detected by indirect immunofluorescence cell-based assay (CBA) or enzyme linked immunosorbent assay (ELISA).

Empirically, the patient was initiated on neurotrophic therapy, including mecobalamin (0.3 mg/day), vitamin B1 (25 mg/day), and vitamin B6 (50 mg/day), administered via intravenous drip, alongside corticosteroid therapy with dexamethasone (5 mg/day, intravenous drip). However, only minimal symptomatic improvement was observed after five days of treatment. In accordance with clinical expert consensus, IVIG therapy was recommended at a dose of 200 mg/kg/day for three days. Unfortunately, the parents of the patient declined this treatment due to financial constraints. Subsequently, efgartigimod, an immune modulator, was proposed as an alternative therapeutic option (7). Detailed informed consent was obtained from the patient’s parents prior to initiation. A trial dose of efgartigimod (10 mg/kg) was administered intravenously. As anticipated, the patient exhibited rapid clinical improvement beginning on the first day post-administration, with 67% resolution of symptoms achieved (SARA_after treatment_=6) by the third day (**Supplementary Table 1**). Follow-up ultrasound examination conducted 14 days after discharge revealed complete resolution of the previously identified left ovarian lesion, which excluded the possibility of ovarian teratomas (**Figure 2**). Unfortunately, the patient has not yet received the second dose of efgartigimod treatment (standard 4-week protocol for MG) due to early clinical resolution (SARA=6 at discharge) and geographic constraints preventing follow-up. This also resulted in the patient not having a repeat brain MRI. However, according to telephone follow-up with the family, the patient’s symptoms have completely relieved and not recurred.

## Discussion

This case report highlights the potential therapeutic role of efgartigimod in the treatment of autoimmune-mediated ACA, particularly in cases refractory to conventional therapies. The patient’s rapid and complete recovery following efgartigimod administration suggests that this agent may be effective in modulating the immune response in antibody-negative ACA, possibly through indirect effects on T and B cell function.

ACA, also known as autoimmune cerebellitis, is a syndrome of cerebellar dysfunction mediated by autoimmune responses (8). Based on its association with tumors, ACA can be classified into paraneoplastic ACA and non-paraneoplastic ACA. The detection of anti-cerebellar antibodies plays a crucial role in the diagnosis of ACA. Diagnosing ACA requires a comprehensive evaluation of clinical manifestations, CSF analysis, neuroimaging, anti-neuronal antibody testing, and related comorbidities (2). In our case, the patient had no significant family history, and screenings for tumors and autoimmune-related antibodies were both negative. Imaging studies also yielded no abnormalities, although an adnexal cyst was incidentally discovered. Additionally, the normal EEG findings and the history of recent menarche helped exclude ovarian teratoma-associated NMDAR encephalitis, which typically presents with prominent psychiatric/behavioral disturbances and seizures. In our case, the patient exhibited prominent cerebellar ataxia as the primary clinical symptom, which led us to consider a diagnosis of seronegative autoimmune cerebellitis. Regarding the EBV serology results, these are most consistent with a past EBV infection. This interpretation is supported by the patient’s report of a mild, self-resolved flu-like illness approximately two weeks prior to presentation. The mild nature and spontaneous resolution of this antecedent illness suggest a relatively low viral load. This may explain the subsequent inability to detect EBV DNA via PCR testing. However, the presence of detectable EBV-specific antibodies (IgG and IgM) is expected, as these immunoglobulins persist in the circulation for a significant period following infection. Critically, EBV infection is a recognized trigger for ACA, which is understood to be a postinfectious autoimmune complication. Literature review indicates that multiple infectious agents, including EBV, cytomegalovirus, influenza virus, varicella-zoster virus and, enteroviruses, have been associated with cerebellar ataxia (9). This evidence suggests that comprehensive pathogen screening should be considered in future patients presenting with comparable neurological presentations to facilitate accurate diagnosis and appropriate management.

However, BBE, though rare in children, was rigorously considered given its characteristic triad of 1) bilateral external ophthalmoplegia, 2) ataxia, and 3) altered consciousness. This postinfectious disorder typically follows respiratory or gastrointestinal infections caused by pathogens including Mycoplasma pneumoniae, Haemophilus influenzae, Campylobacter jejuni, influenza B virus, and cytomegalovirus (10–14). BBE shares pathophysiological features with MFS and GBS, complicating clinical differentiation (13). Critical diagnostic findings absent in our case included: anti-GQ1b/GM1 antibodies and CSF cytoalbuminologic dissociation or pleocytosis (11, 14, 15). While isolated reports describe atypical anti-GD1a antibodies in BBE (10), the detected anti-ganglioside antibodies (anti-GD1a, GT1a, GM4; **Table 2**) lacked diagnostic specificity. Seronegativity for established biomarkers precluded fulfillment of diagnostic criteria. Transiently negative results may reflect early disease stage (<2 weeks), warranting serial assessment in equivocal cases.

Despite corticosteroid administration aligned with established immunotherapy guidelines for immune-mediated cerebellar ataxias (16), no significant improvement was observed. However, following the administration of efgartigimod, the patient’s clinical symptoms rapidly resolved, and the adnexal cyst also disappeared. This suggests that efgartigimod may exert effects similar to conventional IVIG therapy, facilitating the rapid clearance of pathogenic antibodies.

Efgartigimod, as a high-affinity FcRn antagonist, has demonstrated significant efficacy and safety in the treatment of diseases such as MG and CIDP (5, 6). Its mechanism of action, which involves the reduction of circulating IgG levels, has been well-documented in other IgG-mediated autoimmune diseases, including immune thrombocytopenia, pemphigus, and active idiopathic inflammatory myopathy (5). The primary mechanism of efgartigimod involves the reduction or elimination of disease-related antibodies in circulation (5). However, serum IgG levels do not affect the efficacy and safety of efgartigimod. A retrospective study indicated that some autoimmune disease patients did not exhibit elevated serum IgG levels prior to receiving efgartigimod treatment, yet still experienced a reduction in IgG levels and significant clinical symptom improvement (17). Additionally, numerous studies have found that the decrease in IgG levels in patients did not increase the risk of infections, tumors, or other adverse events, suggesting a favorable safety profile (5, 18).

To date, there have been no reported studies on the use of efgartigimod in autoimmune cerebellar ataxia, particularly in pediatric populations. Current research on efgartigimod has predominantly focused on adult populations, with only one case having reported efgartigimod in anti-NMDAR encephalitis in a 16-year-old adolescent (7, 19). Learn from the reported case, we administered efgartigimod to an 11-year-old child with ACA, in whom no pathogenic antibodies were detected. Efgartigimod demonstrated excellent therapeutic efficacy with no apparent adverse effects, a finding consistent with observations in a triple-negative MG patient (20). However, efgartigimod is an off-label use in this case and the safety and possible long-term complications in the pediatric population remain unknown. Additionally, without following the standard dosage protocol for MG (one dose per week for 4 weeks), the patient received only one dose and achieved satisfactory effects and dropped out from the treatment. While emerging evidence supports its use in pediatric autoimmune disorders (7, 19), the dosage and duration of efgartigimod in different diseases and long-term effects require further study. This case provides new insights into the potential role of efgartigimod in the treatment of ACA. Additionally, it suggests that efgartigimod may exert its effects through alternative immune mechanisms in antibody-negative ACA.

This case provides preliminary evidence supporting further investigations into the use of efgartigimod in children with ACA, particularly those who do not respond to standard treatments. Further studies involving pediatric populations are necessary to validate the efficacy and assess the safety profile of efgartigimod. Additionally, the optimal therapeutic dosage and duration of efgartigimod for treating ACA in children remain to be elucidated.

## Conclusion

Autoimmune cerebellar ataxia, while often self-limiting, can present significant diagnostic and therapeutic challenges, particularly in severe or refractory cases. This case report suggests that efgartigimod may offer a promising therapeutic option for pediatric patients with ACA, especially those who do not respond to conventional treatments. Further studies are needed to establish the efficacy, safety, and optimal dosing of efgartigimod in this population.

## Data Availability

The original contributions presented in the study are included in the article/**Supplementary Material**. Further inquiries can be directed to the corresponding authors.
