# Supplementary material for: The utilization of efgartigimod in the treatment of acute cerebellar ataxia: a case report
Source: Front Immunol. 2025 Aug 27;16:1581954. doi: 10.3389/fimmu.2025.1581954 (PMC12420292; doi:10.3389/fimmu.2025.1581954)
Supplement: Supplementary file 1 [file Supplementaryfile1.docx]

**Supplemental Figure 1**


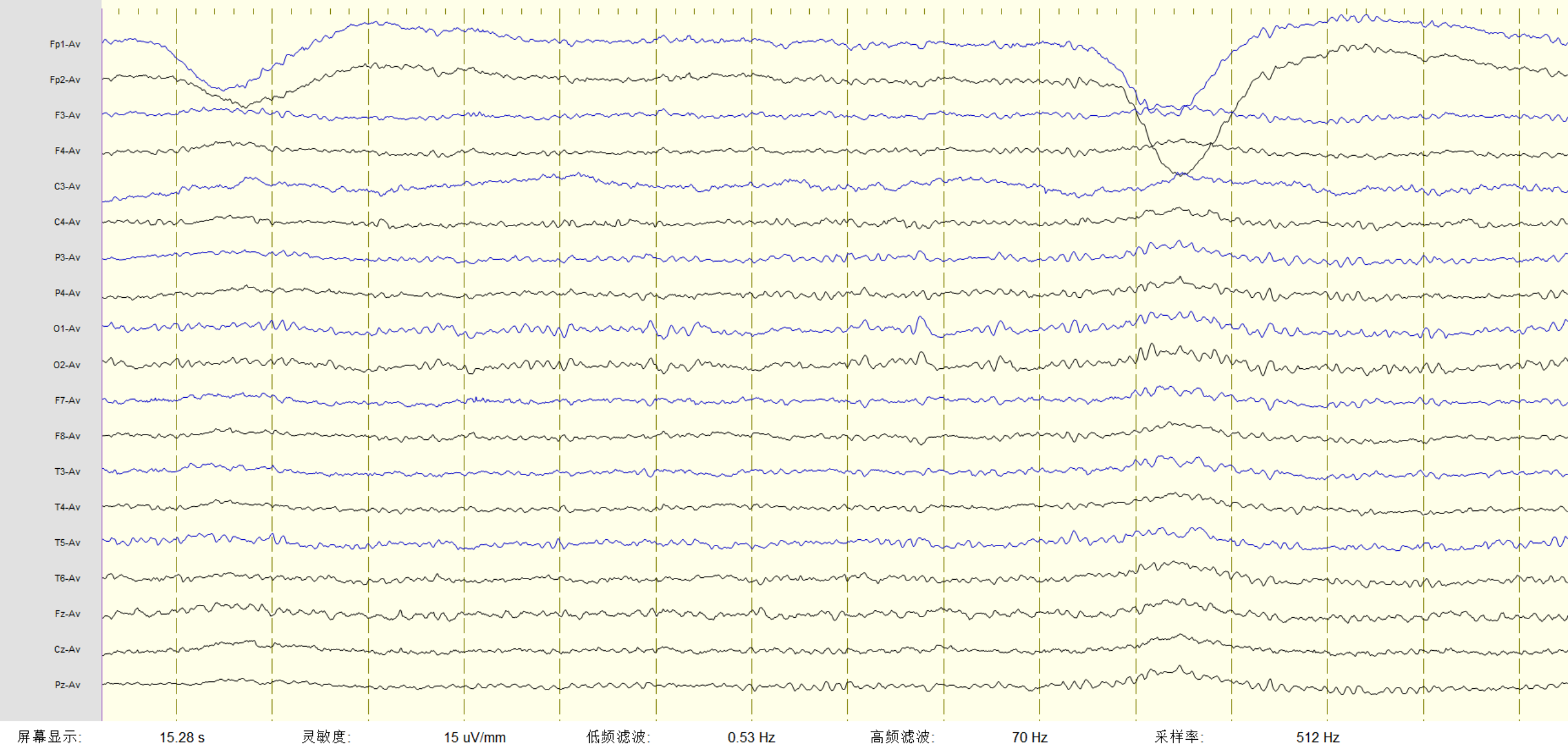


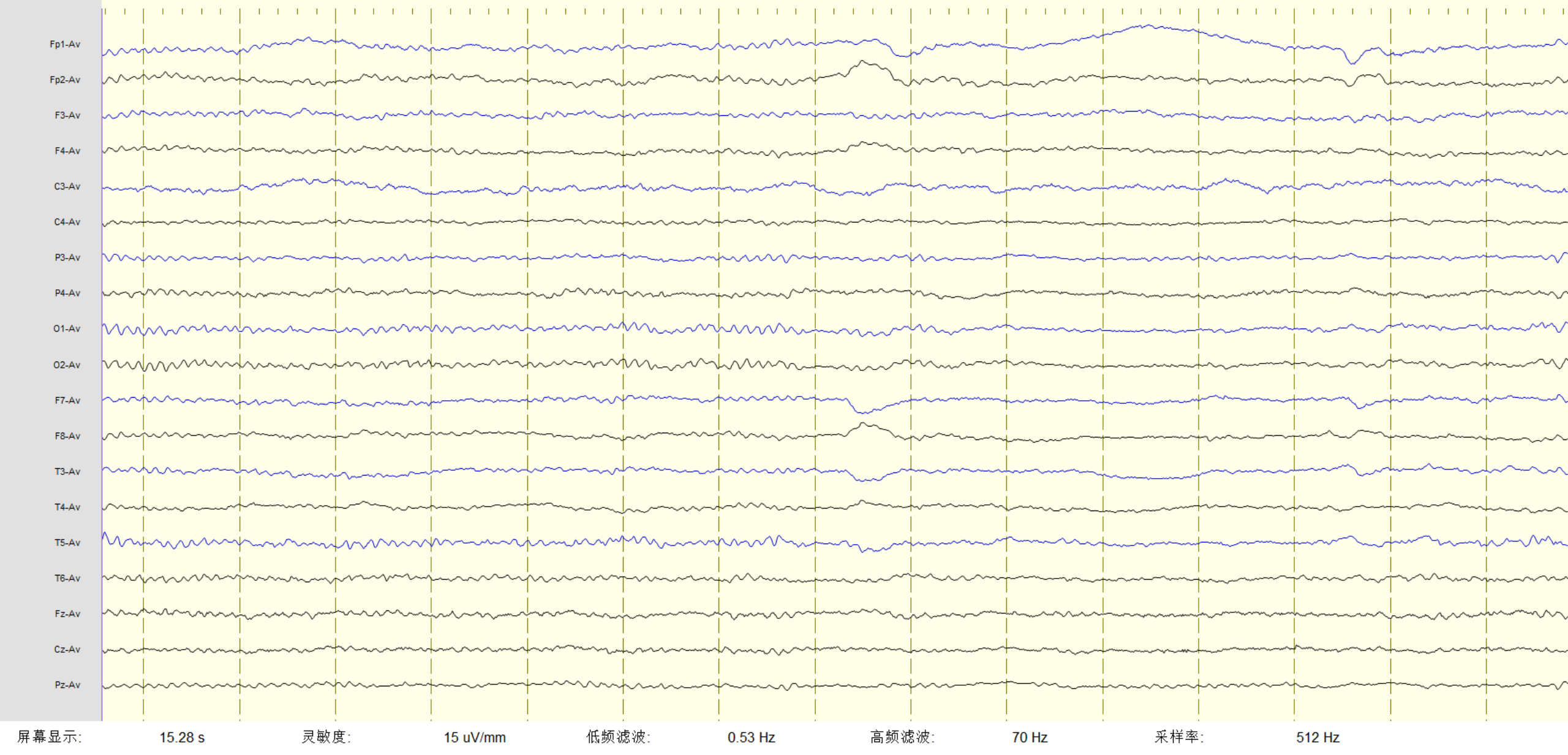


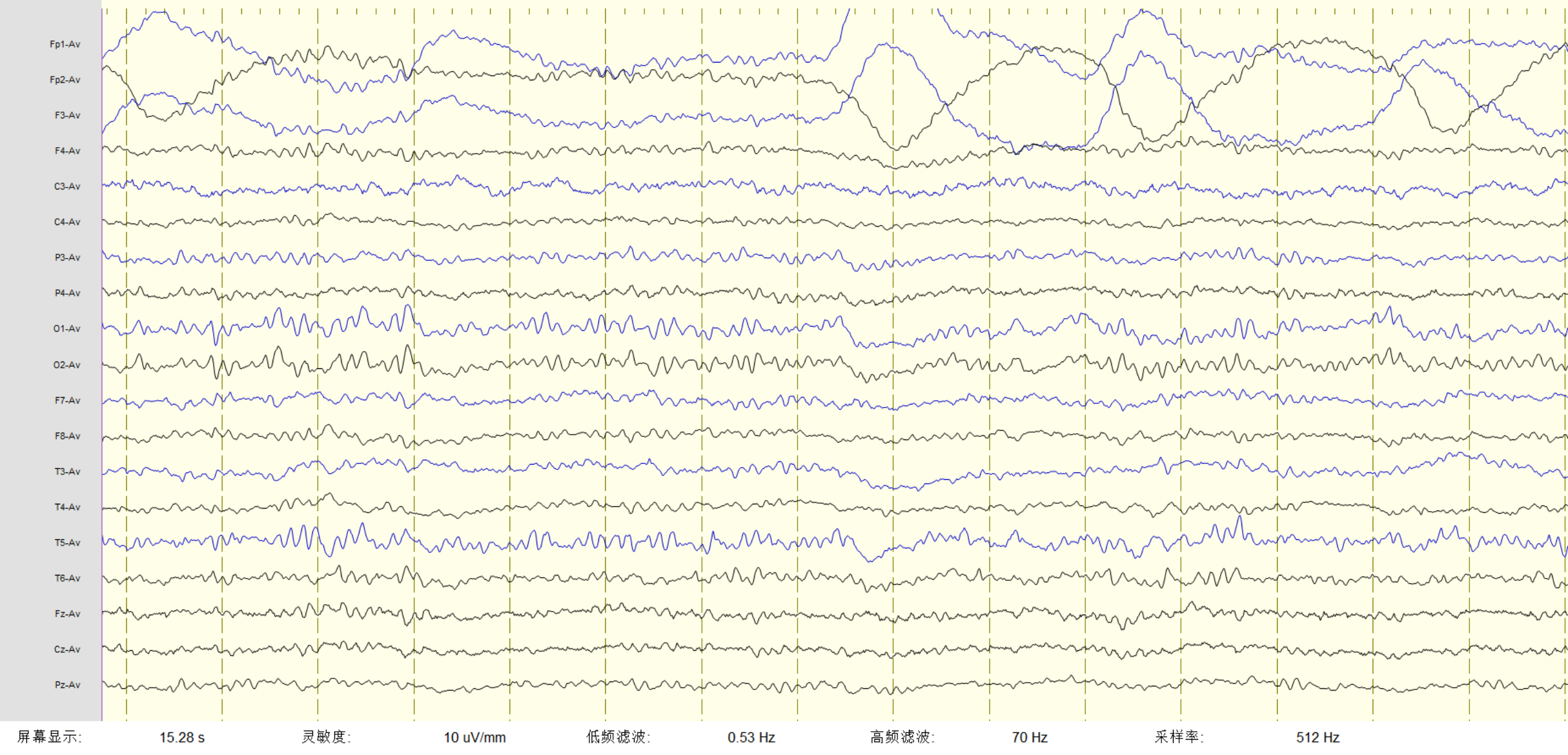


**Supplemental Figure 1**. The EEG results of the patient. EEG = electroencephalogram.
