# Supplementary material for: The utilization of efgartigimod in the treatment of acute cerebellar ataxia: a case report
Source: Front Immunol. 2025 Aug 27;16:1581954. doi: 10.3389/fimmu.2025.1581954 (PMC12420292; doi:10.3389/fimmu.2025.1581954)
Supplement: Supplementary file 2 [file Table1.docx]

| **Supplemental Table 1. Assements for SARA before and after treatment with Efgartigimod.** | | |
| --- | --- | --- |
| **Score** | **Before** | **After** |
| Total | 18 | 6 |
| Gait | 5 | 1 |
| Stance | 3 | 1 |
| Sitting balance | 0 | 0 |
| Speech disturbances | 2 | 1 |
| Finger-chase test | 2 | 1 |
| Nose-finger test | 2 | 1 |
| Fast alternating hand movements | 1 | 0 |
| Heel-shin slide | 3 | 1 |
| SARA were assessed on the first day of admission and three days after efgartigimod. SARA=Scale for the Assessment and Rating of Ataxia. | | |
