# Supplementary material for: The utilization of efgartigimod in the treatment of acute cerebellar ataxia: a case report
Source: Front Immunol. 2025 Aug 27;16:1581954. doi: 10.3389/fimmu.2025.1581954 (PMC12420292; doi:10.3389/fimmu.2025.1581954)
Supplement: Supplementary file 3 [file Table2.docx]

| **Supplemental Table 2. Cerebrospinal fluid (CSF) tests.** | |  |
| --- | --- | --- |
| Test | Result | Reference range (units) |
| Pressure | 120 | 80-180 (mmH_2_O) |
| CSF property | colourless and transparent | |
| Cell count | 1.00 | 10 (*10^6^/L) |
| White cell count | 1 | 10 (*10^6^/L) |
| Protein | 0.11 | 0.15-0.45 (g/L) |
| Glucose | 4.01 | 2.2-3.9 (mmol/L) |
| Chloride | 123.3 | 118-132 (mmol/L) |
| Lactate dehydrogenase (LDH) | 14 | (U/L) |
| Aspartate aminotransferase (AST) | 14 | (U/L) |
| Creatine kinase (CK) | 1 | (U/L) |
| Adenosine deaminase (ADA) | 1 | (U/L) |
| India ink stain | negetive |  |
| Acid-fast stain | negetive |  |
| CSF smear for bacteria | negative |  |
| CSF smear for fungus | negetive |  |
